# Supplementary material for: Beyond 100 THz-spanning ultraviolet frequency combs in a non-centrosymmetric crystalline waveguide
Source: Nat Commun. 2019 Jul 5;10:2971. doi: 10.1038/s41467-019-11034-x (PMC6611800; doi:10.1038/s41467-019-11034-x)
Supplement: Supplementary file 1 — Supplementary information [file 41467_2019_11034_MOESM1_ESM.pdf]

Supplementary Information:

Beyond 100 THz-spanning ultraviolet frequency combs in a non-centrosymmetric crystalline waveguide

Liu et al.

Supplementary Note 1: Phase matching in adiabatic frequency conversion

The wavevector mismatch $\Delta k(\lambda, z)$ between fundamental and second-harmonic (SH) modes in tapered waveguides reads:

$$\Delta k(\lambda, z) = 2k_1(\lambda, z) - k_2(\lambda/2, z) = \frac{4\pi}{\lambda}[n_1(\lambda, z) - n_2(\lambda/2, z)] \tag{1}$$

where λ is the wavelength of the fundamental mode and z is the light propagation distance ($z = 0$ being the initial position). Meanwhile, $k_{1,2}$ and $n_{1,2}$ denote the propagation constants and effective indices relative to the fundamental and SH modes, respectively. In the main text, we show that $\Delta k(\lambda, z)$ can be approximated by the first-order Taylor expansion $\Delta k(\lambda, z) \approx \Delta k(\lambda, z_0) + \Delta z \frac{\partial(\Delta k)}{\partial z}\Big|_{z=z_0}$, where z_0 denotes the perfect phase-matching location with $\Delta k(\lambda, z_0) = 0$. As a result, efficient SH generation with the phase factor $\Delta k(\lambda, z) \cdot \Delta z \approx 0$ can be ensured at a moderate adiabatic interaction length $\Delta z = z - z_0$ [1], as illustrated in Supplementary Fig. 1(a), where an extremely small modulus of $\partial(\Delta k)/\partial z$ is satisfied.

For the linear-tapered geometry in the main text, the waveguide width $w(z) = w_0 - \frac{\Delta w}{L} z$, where $\Delta w = 0.2$ µm equals to the start tapering width $w_0 = 0.5$ µm minus the end width of 0.3 µm, and $L = 5$ mm is the tapering length. As a result, the variation of $\Delta k(\lambda, z)$ along the waveguide, namely $\partial(\Delta k)/\partial z$, can be expressed by:

$$\frac{\partial(\Delta k)}{\partial z} = \frac{\partial(\Delta k)}{\partial \lambda} \frac{d\lambda}{dw} \frac{dw}{dz} \tag{2}$$

Here $d\lambda/dw =$ 0.46 is derived from Fig. 1b of the main text. Therefore, $\partial(\Delta k)/\partial z$ can be calculated when incorporating the simulated $n_{1,2}$ by a finite-element method (FEM) solver. As shown in Supplementary Fig. 1(b), the modulus of $\partial(\Delta k)/\partial z$ ranges from 0.5×10^{-5} to 5×10^{-5} µm^{-2} over the entire bandwidth, suggesting the existence of a relatively large Δz to meet $\Delta k(\lambda, z) \cdot \Delta z \approx 0$. An analytic expression of $\Delta z = \left|\frac{\kappa}{\partial(\Delta k)/\partial z}\right|$ is also derived in Ref. [1], showing a clear dependence of Δz on $\partial(\Delta k)/\partial z$ and the nonlinear coupling coefficient κ. For the chirp-modulated taper waveguide in the main text, Δz is linearly scaled down for each tapered channel because of the reduced tapering length.

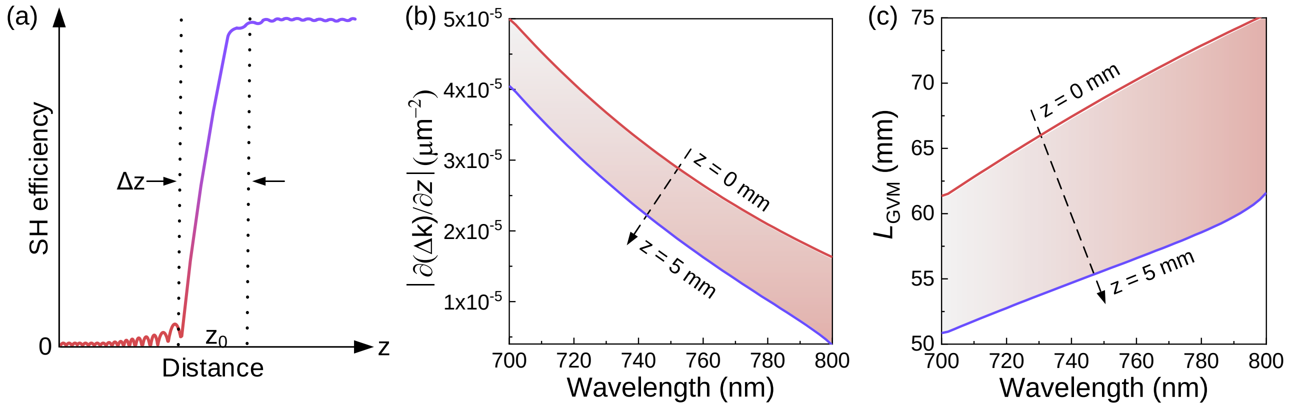

Supplementary Figure 1. Interpretation of adiabatic second harmonic (SH) frequency conversion. **(a)** Sketch of adiabatic SH generation around the phase-matching location z_0 with a interaction length Δz, as discussed in Ref. [1]. **(b)** and **(c)** Calculated modulus of $\partial(\Delta k)/\partial z$ and L_{GVM} of the linear-tapered aluminum nitride (AlN) waveguide versus the wavelength for the propagation distance z from 0 to 5 mm (indicated by the shadow regions).

When the phase-velocity matching discussed above is satisfied, the interacting fundamental and SH modes usually suffer from a group-velocity mismatching (GVM) in nanophotonic waveguides, which is defined as:

$$\mathrm{GVM} = \left|\frac{1}{v_{g1}} - \frac{1}{v_{g2}}\right| \tag{3}$$

where $v_{g1,2}$ are the group velocities of fundamental and SH modes, respectively. The GVM induces a temporal walkoff for the incident fundamental and generated SH pulses when propagating inside the waveguide. After a characteristic length $L_{\mathrm{GVM}} = \tau/\mathrm{GVM}$ (τ being the fundamental pulse duration) [2], the interacting fundamental and SH pulses lose their temporal overlap, resulting in limited conversion efficiency. In our case, the exploited fetomsecond laser delivers a pulse with $\tau = 100$ fs. By incorporating the simulated $v_{g1,2}$ inside the AlN waveguide by FEM, we plot the L_{GVM} in Supplementary Fig. 1(c). It is seen that the derived L_{GVM} is significantly larger than the adiabatic interaction length Δz (below 100 µm) determined by $\partial(\Delta k)/\partial z$ in Supplementary Fig. 1(b). As a result, the influence of the GVM in our adiabatic waveguide is likely to be negligible, which corroborates with the broad bandwidth achieved in the main text.

Supplementary Note 2: Verification of the ultraviolet mode family

To experimentally verify the mode families of generated ultraviolet light in Fig. 2b of the main text, we leverage a modified straight AlN waveguide, whose width (0.44 µm) is intentionally narrowed down to 0.2 µm on one side, as sketched in Supplementary Fig. 2(a). For improved light coupling efficiency, both ends of the waveguide are ultimately tapered to a large width of 3 µm. According to the simulated effective indices in Supplementary Fig. 2(b), the AlN waveguide at a width of 0.44 µm allows the phase macthing between the fundamental transverse magnetic (TM_{00}) mode at 816 nm and the SH TM_{20} modes at 408 nm. Further reducing the waveguide width below 0.28 µm, the ultraviolet TM_{20} mode is cutoff whereas the near-visible TM_{00} mode is still supported. Therefore, the narrowed AlN waveguide acts as a high-order mode filter when the optical pump is introduced from left to right. Supplementary Fig. 2(c) compares the recorded power spectral density (PSD) from the AlN waveguide upon pumping it from different facets. It can be seen that the captured ultraviolet spectra are suppressed by 20 dB when pump is introduced from the left facet, whereas the near-visible spectra remain identical. The result confirms that the generated ultraviolet comb belongs to the TM_{20} mode due to spectral translation through second-harmonic and sum-frequency processes.

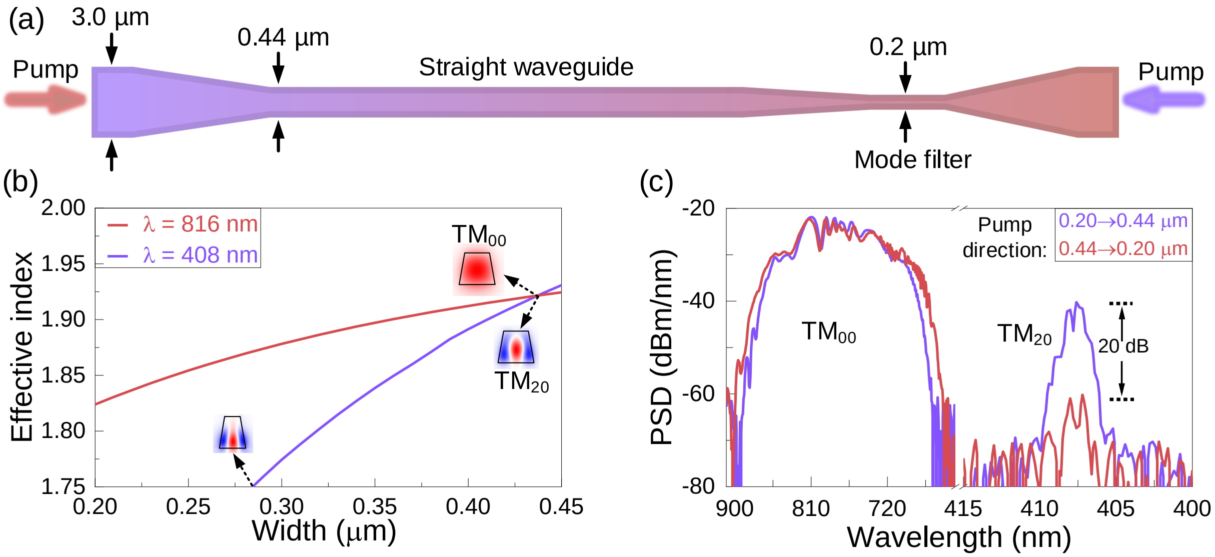

Supplementary Figure 2. Characterization of ultraviolet mode families. **(a)** Illustration of the modified straight AlN waveguide (width of 0.44 µm) with an on-chip mode filter (width of 0.2 µm) on one side so as to block the high-order ultraviolet mode. The red and purple arrows near the waveguide facets indicate different pump directions. **(b)** Simulated effective indices versus the AlN waveguide width (height of 0.5 µm) at the wavelength of 816 and 408 nm by a finite element method (FEM). The phase-matching width is found to be around 0.44 µm for near-visible TM_{00} and ultraviolet TM_{20} modes (electric fields in the inset). **(c)** Experimentally recorded PSD of outgoing light from the AlN waveguide in **(a)** upon feeding the incoming pump pulse from different facets. The legends indicate the pump direction from the width of 0.2 to 0.44 µm or in reverse.

Supplementary Note 3: Calibration of the captured spectral power

To verify the captured PSD in the optical spectrum analyzer (OSA), we extract the average power $P_{\rm ave}$ from the recorded spectra based on the below equation:

$$P_{\rm ave} = \int \mathrm{PSD}(\lambda) d\lambda \tag{4}$$

Then we compare the extracted $P_{\rm ave}$ with the detected one from the optical fiber by a silicon power sensor (Thorlabs S140C). The locations of the optical fiber and the OSA are sketched in Fig. 2(a) of the main text. In Supplementary Fig. 3(a), we show the spectral broadening of the near-visible supercontinuum versus the applied on-chip pump power ($P_{\rm in}$). Supplementary Fig. 3(b) compares the measured $P_{\rm ave}$ from the optical fiber and the extracted $P_{\rm ave}$ from the recorded spectra in the OSA. It is seen that the $P_{\rm ave}$ of the near-visible light in the optical fiber and the OSA differs by ~1.2 dB. Similarly, the extracted $P_{\rm ave}$ of the ultraviolet spectrum in Fig. 2(d) of the main text shows ~2.4 dB deviation with the $P_{\rm ave}$ recorded in the optical fiber. Our result suggests that the power detection using the power sensor and the OSA in our experiment is consistent when accounting for additional insertion loss inside the OSA, especially for the ultraviolet light.

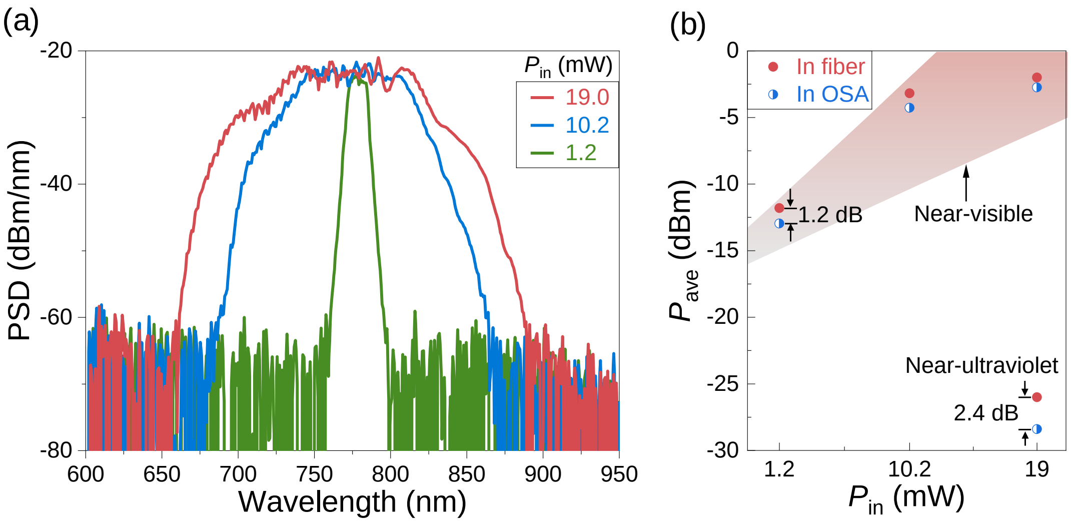

Supplementary Figure 3. Spectral power calibration. **(a)** Recorded spectra of the near-visible supercontinuum from the chirp-tapered AlN waveguide in the main text using a grating-based optical spectrum analyzer (OSA). The pump power $P_{\rm in}$ is estimated from the measured transmittance in Fig. 3(a) of the main text. **(b)** Comparison of the measured (in fiber) and extracted (in OSA) $P_{\rm ave}$ for the near-visible (denoted by the shadow region) and near-ultraviolet lights.

Supplementary references

[1] Suchowski, H., Oron, D., Arie, A. & Silberberg, Y. Geometrical representation of sum frequency generation and adiabatic frequency conversion. *Phys. Rev. A* **78**, 063821 (2008)

[2] Suchowski, H., Porat, G. & Arie, A. Adiabatic processes in frequency conversion. *Laser Photon. Rev.* **8**, 333–367 (2014).
